# Drifting plasmons in two-dimensional electron channels: circuit analogy

**DOI:** 10.1098/rsta.2023.0312

**Published:** 2024-09-09

**Authors:** O. Sydoruk

**Affiliations:** ^1^ Department of Electrical and Electronic Engineering, Imperial College London, London SW7 2AZ, UK

**Keywords:** plasmon, two-dimensional channel, non-reciprocal waveguide, transmission line, instability

## Abstract

Plasmons in two-dimensional electron channels have potential applications in the terahertz frequency range. Equivalent circuit models provide a convenient framework for analysing the plasmons. This article introduces a circuit model for plasmons in the presence of a dc current that flows in a gated channel. It is shown that drifting plasmons can be described by an *LC*-transmission line with distributed dependent sources. A circuit analogue of the Dyakonov–Shur instability is demonstrated. Then, a lumped-element transmission line with dependent sources is analysed, and non-reciprocity is demonstrated for examples of a right- and a left-handed transmission line. Effects of ohmic loss are discussed. The results could be used for the design of non-reciprocal transmission line devices.

This article is part of the theme issue ‘Celebrating the 15th anniversary of the Royal Society Newton International Fellowship’.

## 1. Introduction

It has been known at least since the 1960s that plasma waves can interact with dc currents in semiconductors [1,2]. Early investigations were partially motivated by the success of vacuum travelling-wave devices. For example, the solid-state travelling-wave amplifier proposed by Solymar & Ash [3] was an analogue of the travelling-wave tube and relied on the interaction between drifting plasmons and an artificial slow-wave structure. Although practical levels of gain have not yet been achieved, the device proposal is still attracting interest [4,5]. Contemporary investigations of drifting plasmons are often motivated by their potential for devices in the terahertz (THz) frequency range, notably oscillators and amplifiers [6–10]. Growing attention is also being paid to other non-reciprocal effects arising from the electron drift, for example, enhanced nonlinear optical interactions [11] and non-reciprocal radiative heat transfer [12].

Travelling-wave interactions between drifting plasmons and slow waves were the subject of the Newton Fellowship the author held in 2009–2011. After an investigation of the effect of electron collisions on plasma instabilities [4], optical plasmons were considered as potential slow waves [13,14]. Afterwards, alternative instabilities that do not require high electron drift velocities were studied, in particular, an analogue of the Dyakonov–Shur instability in field-effect transistors with the Corbino geometry [15]. This work led to a development of a modal analysis for drifting plasmons in two-dimensional electron channels [6,7,16–20].

Rigorous theoretical modelling of plasmons requires one to solve Maxwell’s equations coupled to equations that describe the electron dynamics. Often, such an analysis can only be done by time-consuming numerical simulations [21,22], which may lack the insight of analytical results. Naturally, approximate analytical models of plasmons have attracted much attention. Such models typically describe the electron dynamics by hydrodynamic equations of motion [23,24]. On the other hand, a common way to simplify electromagnetic problems is by using equivalent circuit models, which have been employed for plasmons, for example, in [25–27].

Circuit models of plasmons have so far concentrated on devices without dc currents. This article discusses circuit modelling of drifting plasmons and then uses the circuit analogy to introduce non-reciprocal transmission lines. Section 2 reviews a standard description of plasmons in two-dimensional channels. Section 3 introduces a circuit model for drifting plasmons. Section 4 considers a circuit analogue of the Dyakonov–Shur instability. Section 5 applies the approach to non-reciprocal transmission lines. Section 6 draws conclusions.

## 2. Plasmons in gated two-dimensional channels

**Figure 1.**
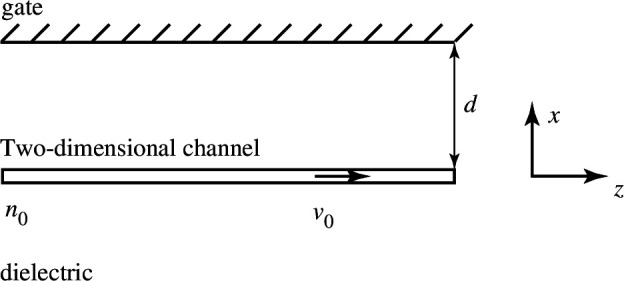
Schematic representation of a gated two-dimensional channel supporting plasmons. A dc current flowing along the channel modifies the plasmon properties.

A typical geometry supporting plasmons consists of a two-dimensional electron channel embedded in a uniform dielectric, see figure 1. The channel is at x=0 and is unbound along the y-axis. A conducting gate is laid over the channel at a distance d. A dc voltage that is applied between the channel and the gate controls the electron density $n_{0}$ in the channel. A dc voltage applied to the channel excites a dc current with a density $J_{0}=en_{0}v_{0}$, where e is the electron charge and $v_{0}$ is the electron drift velocity.

Neglecting retardation, time-varying electromagnetic fields can be described by an electrostatic potential φ. The corresponding time-varying electron density is n, the electron velocity is v and the current density is J. If the amplitudes of the time-varying components are much smaller than their dc counterparts, the expression for the time-varying electron current density can be linearized as


(2.1) $$J=en_{0}v+env_{0}$$

and the hydrodynamic equation of motion (the Euler equation) can be linearized as


(2.2) $$\frac{\partial v}{\partial t}+v_{0}\frac{\partial v}{\partial z}=-\frac{e}{m}\frac{\partial \varphi }{\partial t},$$

where m is the effective electron mass and a spatial variation along the z-axis is assumed. The current density and the electron density obey the linearized continuity equation in the form


(2.3) $$\frac{\partial J}{\partial z}+e\frac{\partial n}{\partial t}=0.$$

If the gate-to-channel distance d is small, the electric field of the plasmon between the channel and the gate can be taken as being spatially uniform in the x-direction so that the channel and the gate form a capacitor. If the capacitance per unit area is C, the time-varying potential can then be written as


(2.4) $$\varphi =\frac{en}{C}.$$

Equations (2.2) and (2.3) can be rewritten for the current and the potential, using (2.1) and (2.4) as


(2.5) $$\frac{\partial J}{\partial t}-2v_{0}C\frac{\partial \varphi }{\partial t}=-\left(\frac{e^{2}n_{0}}{m}-v_{0}^{2}C\right)\frac{\partial \varphi }{\partial z}\;\frac{\partial J}{\partial z}+C\frac{\partial \varphi }{\partial t}=0.$$

**Figure 2.**
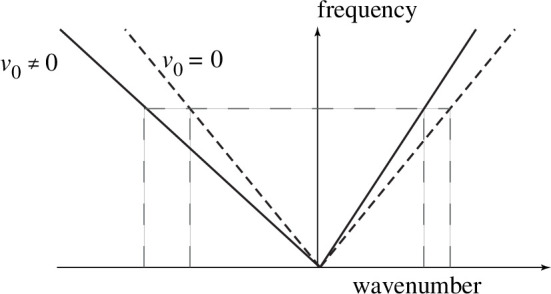
In the absence of a dc current, the dispersion curves (dashed lines) of the counter-propagating plasmons are identical. They are different in the presence of a dc current (solid lines).

For a harmonic time variation, solutions can be sought in terms of waves (plasmons) for which all quantities vary as exp⁡[j(ωt−kz)]. Here, ω is the angular frequency and k is the wavenumber. Then, (2.5) can be written as


(2.6) $$\omega \tilde{J}-2\omega v_{0}C\tilde{\varphi }=\left(\frac{e^{2}n_{0}}{m}-v_{0}^{2}C\right)k\tilde{\varphi }\;k\tilde{J}=\omega C\tilde{\varphi },$$

where $\tilde{J}$ and $\tilde{\varphi }$ denote the amplitudes of the current density and the potential. Non-trivial solutions exist when plasmons obey the dispersion relation in the form


(2.7) $$\omega =(v_{0}\pm s)k,$$

where $s^{2}=e^{2}n_{0}/(mC)$. A striking feature of the two-dimensional channel, as evidenced by the dispersion relation (2.7), is that it acts as a non-reciprocal plasmonic waveguide in the presence of the electron drift. At the same frequency, the counter-propagating drifting plasmons have different wavenumbers, whereas the wavenumbers are the same in the absence of drift, see figure 2.

## 3. Circuit model for plasmons

**Figure 3.**
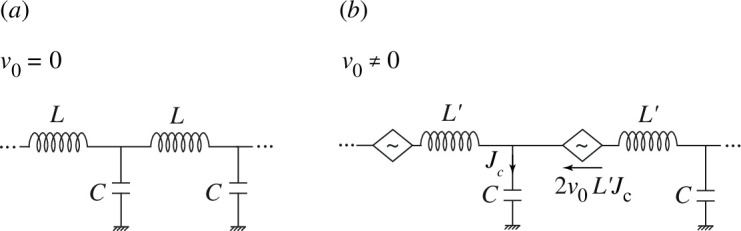
The standard LC transmission line (*a*) can describe plasmon propagation in two-dimensional channels in the absence of dc currents. The effect of a dc current can be described by a current-controlled voltage source (*b*).

This section considers equivalent circuit models for plasmons. Assuming no dc current, $v_{0}=0$, (2.5) take the form


(3.1) $$L\frac{\partial J}{\partial t}=-\frac{\partial \varphi }{\partial z}\;C\frac{\partial \varphi }{\partial t}=-\frac{\partial J}{\partial z},$$

where $L=m/(e^{2}n_{0})$ is the per-unit-area kinetic inductance. Equations (3.1) are in the form of the standard transmission-line equations. Figure 3*a* shows the equivalent circuit, which has also been used for modelling two-dimensional plasmons in the absence of drift [25–27].

In the presence of a dc current, the second equation in (3.1) remains unchanged, and the term C∂φ/∂t has the meaning of the current $J_{c}$ that flows through the capacitor C. Therefore, the first of equations (2.5) can be rewritten in the form


(3.2) $$L^{\prime }\frac{\partial J}{\partial t}-2v_{0}L^{\prime }J_{c}=-\frac{\partial \varphi }{\partial z},$$

where $L^{\prime }=1/(e^{2}n_{0}/m-v_{0}^{2}C)$ is a modified inductance, and it is assumed that $v_{0}<s$. The term $2v_{0}L^{\prime }J_{c}$ has the meaning of a voltage and can be represented by a current-controlled voltage source whose output is proportional to the capacitor current and to the electron drift velocity. Figure 3*a* shows the resulting equivalent circuit for the two-dimensional channel in the presence of a dc current.

## 4. Circuit analogue of Dyakonov–Shur instability

The circuit equations that describe the transmission line of figure 3*b* are the same as the equations for the plasmon potential and current density, (2.5), and therefore, the same phenomena will be observed both in the gated electron channel and the transmission line. In particular, the expressions for the current and voltage in an infinitely long transmission line under a harmonic excitation will take the form


(4.1) $$\varphi =\tilde{\varphi }e^{j(\omega t-kz)}\;\tilde{J}=J_{0}e^{j(\omega t-kz)}\;,$$

and the dispersion relation is of the form


(4.2) $$k_{1,2}=\frac{\omega }{v_{0}\pm \sqrt{\frac{1}{L^{\prime }C}+v_{0}^{2}}}=\frac{\omega }{v_{0}\pm s}.$$

The transmission line supports two counter-propagating waves with different wavenumbers. Despite the presence of active elements, the wavenumbers are real-valued, and the waves propagate along the line with a constant amplitude.

On the other hand, a finite length of the transmission line short-circuited at one end and open-circuited at the other will exhibit an analogue of the Dyakonov–Shur instability. In a classical work [23], Dyakonov & Shur studied drifting plasmons in the channel of a field-effect transistor. Their model comprised the same channel as shown in figure 1, but the channel had a finite length and was terminated by a source and a drain. They showed that plasmons can become unstable (their amplitude grows in time) if asymmetric boundary conditions are realized at the source and the drain. The transmission line provides a circuit perspective on the effect. Assuming a line of a length l and omitting the common multiplier exp⁡(jωt), the current and voltage are given by a superposition of two counter-propagating waves in the form


(4.3) $$J(z)=J_{1}e^{-jk_{1}z}+J_{2}e^{-jk_{2}z}\;\varphi (z)=\frac{1}{\omega C}\left(k_{1}J_{1}e^{-jk_{1}z}+k_{2}J_{2}e^{-jk_{2}z}\right).$$

If the line is open-circuited at the end ($J{|}_{z=l}=0$), the input impedance takes the form


(4.4) $$Z_{in}=\frac{\varphi {|}_{z=0}}{J{|}_{z=0}}=\frac{1}{2\omega C}\left[(k_{1}+k_{2})-j(k_{1}-k_{2})\cot\frac{(k_{1}-k_{2})l}{2}\right]=-v_{0}L^{\prime }-jsL^{\prime }\cot(\omega sL^{\prime }Cl).$$

The real part of the input impedance, $-v_{0}L^{\prime }$, is negative for $v_{0}>0$. Therefore, an open-circuited transmission line is a negative-resistance device. Assuming now that the line is short-circuited at the input, as per the Dyakonov–Shur boundary conditions, the complex-valued frequency $\omega =\omega^{\prime }-j\omega^{\prime \prime }$ can be found from the condition $Z_{\mathrm{in}}=0$ or


(4.5) $$v_{0}+js\;\cot(\omega sL^{\prime }Cl)=0,$$

which yields the known expressions for the frequency and the increment of the Dyakonov–Shur instability [23],


(4.6) $$\begin{array}{l} \begin{array}{rl} & \omega^{\prime }=\frac{1}{2sL^{\prime }Cl}\pi n=\frac{s^{2}-v_{0}2}{2sl}\pi n\;,\;n=1,2,...\\ & \omega^{\prime \prime }=\frac{1}{sL^{\prime }Cl}atanh\frac{v_{0}}{s}=\frac{s^{2}-v_{0}^{2}}{2sl}\;\ln\frac{s+v_{0}}{s-v_{0}} \end{array} \end{array}.$$

## 5. Non-reciprocal transmission lines

**Figure 4.**
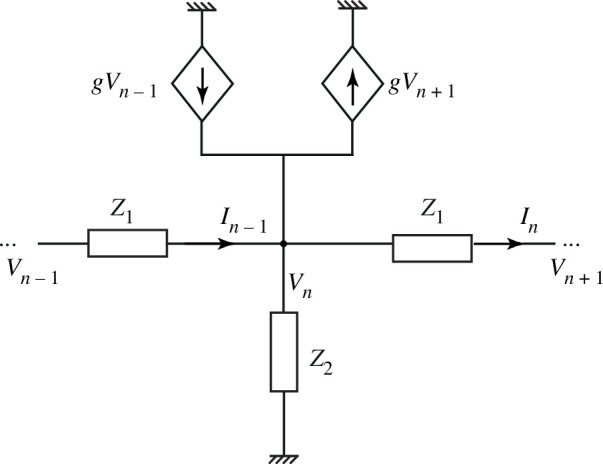
A lumped-element non-reciprocal transmission line with voltage-controlled current sources.

The circuit model of §3 links two-dimensional channels to non-reciprocal circuits with active elements [28,29], and the approach can be used to devise lumped-element non-reciprocal transmission lines with different topologies. Figure 4 shows an example. Here, $Z_{1}$ and $Z_{2}$ are two impedances that form a unit cell of an infinitely long line. Two additional currents are injected into node n by voltage-controlled sources. These currents flow in the opposite directions, and are determined by the voltages in the neighbouring nodes $V_{n\pm 1}$ and by the transconductance g.

The circuit can be analysed by the standard approach of applying Kirchhoff’s laws and assuming wave solutions, as follows. The circuit equations for node n are


(5.1) $$I_{n}=\frac{V_{n}-V_{n+1}}{Z_{1}}$$

and


(5.2) $$I_{n-1}-I_{n}-\frac{V_{n}}{Z_{2}}+gV_{n-1}-gV_{n+1}=0.$$

Substituting (5.1) into (5.2) yields


(5.3) $$\frac{V_{n-1}-V_{n+1}-2V_{n}}{Z_{1}}-\frac{V_{n}}{Z_{2}}+g(V_{n-1}-V_{n+1})=0.$$

Assuming wave solutions in the form $V_{n}=\tilde{V}e^{-j\kappa n}$, where *κ* is the phase change per unit cell, yields the following dispersion relation


(5.4) $$\cos\kappa +jgZ_{1}\sin\kappa =1+\frac{Z_{1}}{2Z_{2}}.$$

Assuming both $Z_{1}$ and $Z_{2}$ have imaginary values (as they do for ideal transmission lines), real-valued solutions for κ are possible. These are in the form


(5.5) $$\kappa =atan(jgZ_{1})\pm acos\frac{1+\frac{Z_{1}}{2Z_{2}}}{\sqrt{1-(gZ_{1}{)}^{2}}}.$$

The non-reciprocal nature of the waves described by (5.4) can be seen more clearly by considering an approximate solution, assuming κ≪1 and $g\ll |1/\sqrt{-Z_{1}Z_{2}}|$. Then (5.4) takes the form of a quadratic equation


(5.6) $$\kappa^{2}-2jgZ_{1}\kappa +\frac{Z_{1}}{Z_{2}}=0,$$

whose approximate solutions are


(5.7) $$\kappa =\pm \sqrt{-\frac{Z_{1}}{Z_{2}}}+jgZ_{1}.$$

If g=0, (5.7) gives two opposite values of κ, corresponding to two counter-propagating waves supported by a reciprocal line. However, if g≠0, the positive and negative solutions of (5.7) do not have equal absolute values, so that the two counter-propagating waves have different values of phase change per unit cell.

**Figure 5.**
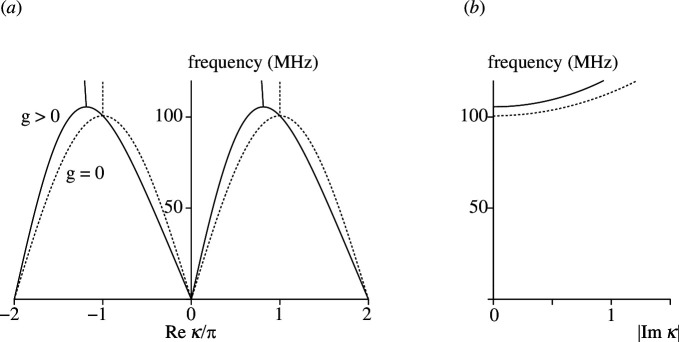
Dispersion diagram (propagation constant (*a*) and attenuation constant (*b*)) for the right-handed reciprocal (dashed) and non-reciprocal (solid) transmission lines.

Several illustrative numerical examples are considered next. In the first example, $Z_{1}=j\omega L$ and $Z_{2}=1/(j\omega C)$. Without the dependent sources, it is a standard right-handed lumped-element transmission line. It acts as a lossless low-pass filter with the cut-off frequency of $1/(\pi \sqrt{LC})$. Consequently, the wavenumbers are real-valued below the cut-off. Figure 5 shows the dispersion diagrams assuming L=100 nH, C=100 pF and for two values of the transconductance, g=0 (dashed lines) and g=10 mS (solid lines). Figure 5*a* shows the frequency dependence of the real part of the wavenumber in two Brillouin zones and figure 5*b* shows the corresponding frequency dependence of the absolute value of the imaginary part of the wavenumber.

When g=0, the dispersion curves are identical for both propagation directions, and show the expected low-pass behaviour with the cut-off frequency of 100 MHz. Below the cut-off frequency, the wavenumbers are real-valued. Above the cut-off frequency, the imaginary part of the wavenumbers is non-zero showing exponential decay of the wave amplitude away from a source, and the real part of the wavenumbers is equal to ±π, showing that the sign of the wave amplitude alternates from unit cell to unit cell. When g>0 (solid lines in figure 5), the counter-propagating waves are no longer identical to each other. The low-pass behaviour of the circuit is retained, with purely real wavenumbers below a cut-off frequency. The cut-off frequency has increased somewhat compared with the case of g=0, and the wavenumbers in the stop-band are now complex-valued.

At low frequencies, the behaviour of the dispersion curves in figure 5 is the same as for the plasmon dispersion curves, see figure 2. The approximate dispersion relation (5.7) yields


(5.8) $$\omega =\kappa \left(\frac{g}{C}\pm \frac{1}{\sqrt{LC}}\right)$$

and its form is identical to that of (2.7).

**Figure 6.**
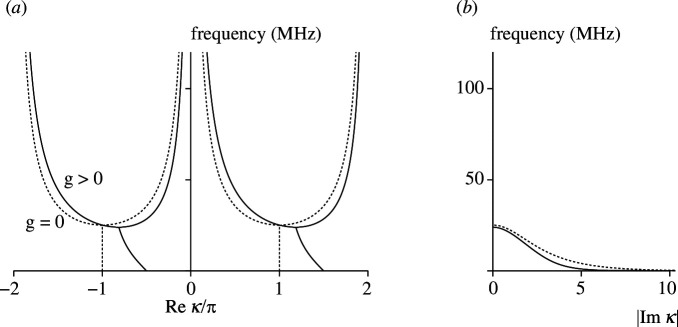
Dispersion diagram (propagation constant (*a*) and attenuation constant (*b*)) for left-handed reciprocal (dashed) and non-reciprocal (solid) transmission lines.

In the next example, the inductances and capacitances are interchanged in the transmission line, so that $Z_{1}=1/(j\omega C)$ and $Z_{2}=j\omega L$. It is an example of a left-handed transmission line. In the absence of the dependent sources, g=0, it has a high-pass behaviour with the cut-off frequency of $1/(4\pi \sqrt{LC})$. Figure 6 shows the dispersion relations, using the same nomenclature as in the previous example of the right-handed transmission line. For g=0, the transmission line is reciprocal; the wavenumbers are real-valued above the cut-off frequency of around 25 MHz. For g>0, the transmission line is non-reciprocal. The wavenumbers remain real-valued above a cut-off frequency, whose value has decreased somewhat compared with the reciprocal line. In the stop-band, the wavenumbers are complex-valued.

**Figure 7.**
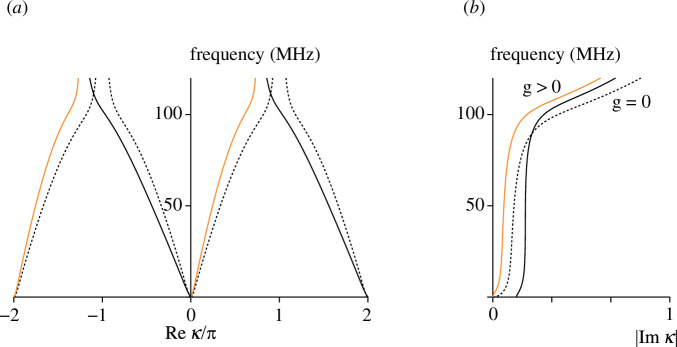
Dispersion diagram (propagation constant (*a*) and attenuation constant (*b*)) for lossy right-handed reciprocal (dashed) and non-reciprocal (solid) transmission lines.

The next example considers the effect of ohmic loss. The transmission line is the same as the one in figure 5, but a resistance R has now been added in series with the inductor, so that $Z_{1}=j\omega L+R$ and $Z_{2}=1/(j\omega C)$. The availability of low-loss components in the MHz-frequency range suggests that R can be low in practice. However, to draw analogies with lossy plasmons, high values of R are of interest. Figure 7 shows the dispersion curves assuming R=10 Ω. When g=0 (dashed black lines), the transmission line is reciprocal, but the wavenumbers are complex-valued both in the pass- and in the stop-band. As a result, out-of-band propagation is permitted, but the loss is high. When g>0 (solid orange and black lines), the two counter-propagating waves have different real and imaginary parts of the wavenumbers. The imaginary part of the wavenumber for the faster wave (whose dispersion is shown by the orange lines) is lower than both for the other wave and for the waves with g=0. The existence of lower-loss and higher-loss waves along the transmission line is analogous to the plasmon propagation in the presence of electron collisions (e.g. [17]).

It is known that drifting solid-state plasmas can support waves even in the collision-dominated regime, in which ωτ≪1 (τ is the electron collision time). Such waves can propagate only in one direction, determined by the electron drift velocity, and their dispersion relation is $\omega =kv_{0}$. A transmission-line analogue can be constructed as follows. Assuming an infinitely large resistance in the series branch of the transmission line of figure 4, $I_{n}=I_{n-1}=I_{n+1}=0$, so that (5.2) becomes


(5.9) $$\frac{V_{n}}{Z_{2}}+gV_{n+1}-gV_{n-1}=0.$$

Assuming $Z_{2}=1/(j\omega C)$ yields a dispersion relation in the form


(5.10) $$\sin\kappa =\frac{\omega C}{2g}.$$

Similar to solid-state plasmas, the waves propagate only in one direction, determined by the sign of g.

## 6. Conclusions

The analysis showed that plasmons propagating in gated two-dimensional electron channels can be described by LC-transmission lines that are loaded periodically by current-controlled voltage sources. In an infinitely long line, the effect of the sources is to change the wavenumbers of counter-propagating waves, leading to non-reciprocal propagation. However, in a resonator that models a field-effect transistor under the Dyakonov–Shur boundary conditions, the presence of dependent sources gives rise to a non-zero real part of the impedance whose sign is determined by the direction of the electron drift. Negative resistance corresponds to the regime of the Dyakonov–Shur instability.

A lumped-element transmission line with dependent sources was then analysed. The unit cell contained two current sources that were controlled by the voltages in the two neighbouring unit cells. Despite the use of active elements, stable wave propagation was demonstrated, characterized by real-valued wavenumbers in the passbands of a lossless line. The main effect of the sources was to alter the propagation constants, rendering them unequal for the two counter-propagating waves. Non-reciprocity was demonstrated for two examples of right- and left-handed transmission lines. Effects of ohmic losses were considered and analogies with lossy plasmons were drawn. The results could be useful for the design of active non-reciprocal transmission-line devices.

## Data Availability

This article has no additional data.
